# A case of periosteal fasciitis located in the mandible in a child

**DOI:** 10.1007/s11282-021-00544-4

**Published:** 2021-06-18

**Authors:** Sato Eida, Yuka Hotokezaka, Miho Sasaki, Hitoshi Hotokezaka, Shuichi Fujita, Ikuo Katayama, Yukinori Takagi, Misa Sumi

**Affiliations:** 1 Department of Radiology and Biomedical Informatics, Nagasaki University Graduate School of Biomedical Sciences, 1-7-1 Sakamoto, Nagasaki, 852-8588 Japan; 2 Department of Clinical Oral Oncology, Nagasaki University Graduate School of Biomedical Sciences, 1-7-1 Sakamoto, Nagasaki, 852-8588 Japan; 3 Department of Orthodontics and Dentofacial Orthopedics, Nagasaki University Graduate School of Biomedical Sciences, 1-7-1 Sakamoto, Nagasaki, 852-8588 Japan; 4 Department of Oral Pathology, Nagasaki University Graduate School of Biomedical Sciences, 1-7-1 Sakamoto, Nagasaki, 852-8588 Japan

**Keywords:** Periosteal fasciitis, Mandible, Child, Magnetic resonance imaging, 18F-fluorodeoxyglucose positron-emission tomography/computed tomography

## Abstract

Periosteal fasciitis (PF), a subtype of nodular fasciitis, is an uncommon benign soft-tissue mass that originates from the periosteum or tissues adjacent to bones. PF has rarely seen in children, especially involving in the mandible. This case report presents a rare case of PF originating from the periosteum of the mandible in an 11-year-old girl. She was referred to our hospital with fast-growing painless swelling in her left mandible. Computed tomography revealed an exophytic juxtacortical mass eroding the lower part of the left mandible and lower mandibular cortex with a periosteal reaction. The mass showed low signal intensity on T1-weighted magnetic resonance imaging (MRI) and high signal intensity on T2-weighted MRI. The apparent diffusion coefficient (ADC) of the lesion found to be moderate. Dynamic contrast-enhanced MRI revealed a gradual increment pattern in the central region of the mass. On 18F-fluorodeoxyglucose (FDG) positron-emission tomography/computed tomography (PET/CT), relatively high 18F-FDG uptake was observed on the early scan and the 18F-FDG uptake was declined on the delayed scan. The clinical and conventional radiological findings of the mass were suggestive of malignancy. However, the findings of ADC and dynamic MRI and dual-time-point FDG-PET/CT favored benign etiology over malignant etiology. Histological and immunohistochemical findings along with reactive ossification of the periosteum confirmed the diagnosis of PF. Currently, comprehensive examinations, such as clinical, imaging, and histopathological examinations, are recommended for the definitive diagnosis of PF, while MRI and dual-time-point FDG-PET/CT could have a potential usefulness to differentiate from malignancy.

## Introduction

Nodular fasciitis (NF) is a rare and benign soft-tissue mass often misdiagnosed as a malignant neoplasm because of its fast and infiltrative growth pattern. It is considered a reactive process that involves the proliferation of mesenchymal origin cells, such as fibroblasts and myofibroblasts; however, the pathogenesis of NF remains unknown [1]. NF was first described as pseudosarcomatous fasciitis by Konwaler et al. in 1955 [2]. It is usually found in the upper extremities, followed by the trunk, head, neck, and lower extremities, and is most common in adults aged 20–40 years [3]. Its prevalence in children is low, accounting for only 10% of reported cases. In the pediatric population, although NF is most commonly reported to occur in the head and neck, its location may vary [4].

NF demonstrating a direct association with bone is termed periosteal fasciitis (PF). PF is a rare, benign, self-limiting condition that originates from the periosteum or tissues adjacent to bones and is considered a subtype of NF. It exhibits histological features similar to subcutaneous NF, constituting approximately 4% of all NF cases [1]. Additionally, PF is related to reactive periosteal overgrowth with reactive bone formation or bone destruction [3, 4]. Only a few articles have been published on PF in pediatric patients. These articles have indicated that PF most commonly occurs in the head and neck area and rarely in the mandible [1].

The imaging features of NF are non-specific and variable. Although NF is generally seen as a relatively well-defined, soft-tissue mass of superficial location on computed tomography (CT) and magnetic resonance imaging (MRI), there was no definitive image pattern for the diagnosis of NF in previous studies [4–6]. Also, 18F-fluorodeoxyglucose (FDG) positron-emission tomography/computed tomography (PET/CT) shows high 18F-FDG uptake in NF, thus making it difficult to distinguish NF from malignant tumors. Since PF shows bone formation or bone destruction in addition to the imaging features of NF, an evidence of bone involvement on imaging along with high 18F-FDG uptake may lead to concerns for malignant neoplasms [7, 8]. Currently, comprehensive examinations, such as clinical, imaging, and histopathological examinations, are recommended for the definitive diagnosis of PF, while the usefulness of imaging in the diagnosis of PF remains controversial.

We report a case of PF in the mandible in an 11-year-old girl. To the best of our knowledge, pediatric cases of PF located in the mandible are rare, and this report may be the third report. In addition, we present and discuss the potential usefulness of MRI and dual-time-point FDG-PET/CT features in this case, to distinguish PF from malignant tumors, and eventually, in the diagnosis of PF.

## Case report

**Fig. 1:**
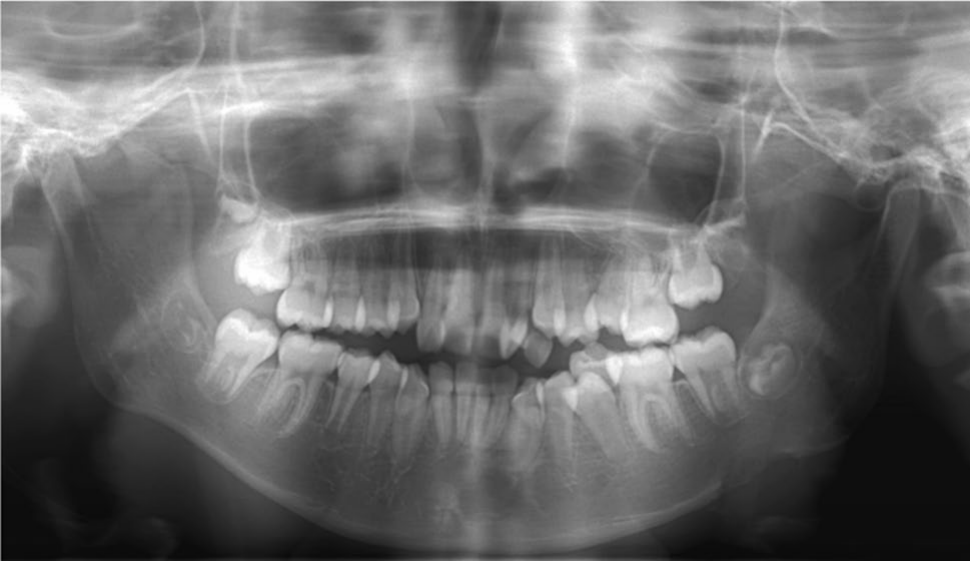
Panoramic radiography of the lesion in the left mandible. Panoramic radiography showing a rounded radiolucent lesion with irregular border

An 11-year-old girl was referred to our hospital for further examination and treatment of swelling in the left mandible. She noticed a rapidly growing painless swelling in the left mandible over 2 weeks. Physical examination revealed a hard mass in the left mandible with no pain or signs of acute inflammation. She had no history of trauma or other diseases. Panoramic radiography revealed a rounded radiolucent lesion with irregular border (Fig. 1). Because the clinical and panoramic radiographic findings of the lesion were suggestive of tumor, especially malignancy, CT, MRI and FDG-PET/CT were performed subsequently.

**Fig. 2:**
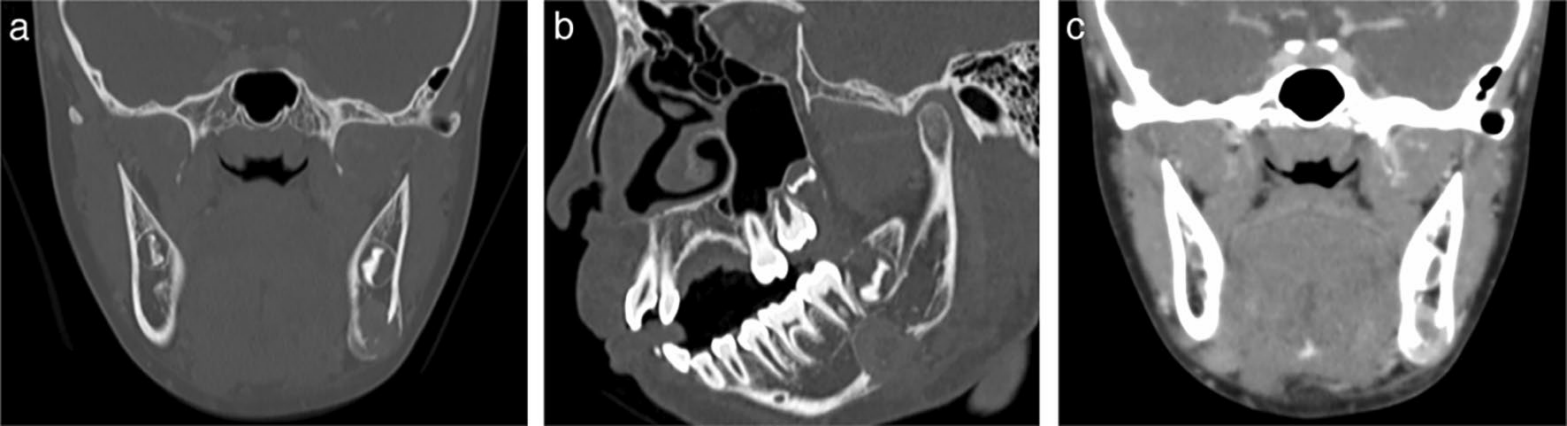
Computed tomography (CT) imaging of the lesion in the left mandible. CT showing an exophytic juxtacortical mass eroding the cortical bone of the left mandible with bone window settings (window width: 3000 HU; window level: 600 HU). Note the periosteal reaction outside the lesion. **a** Coronal image. **b** Sagittal image. **c** Coronal contrast-enhanced image showing weak contrast enhancement inside the lesion

CT revealed a 1.7 × 1.8 × 1.6 cm exophytic juxtacortical mass eroding the cortical bone (Fig. 2a, b). The mass absorbed the lower part of the left mandible and lower mandibular cortex. Furthermore, a periosteal reaction was detected outside the lesion. Contrast-enhanced CT revealed that although most of the lesion was well enhanced, the area inside the lesion was weakly enhanced (Fig. 2c).

**Fig. 3:**
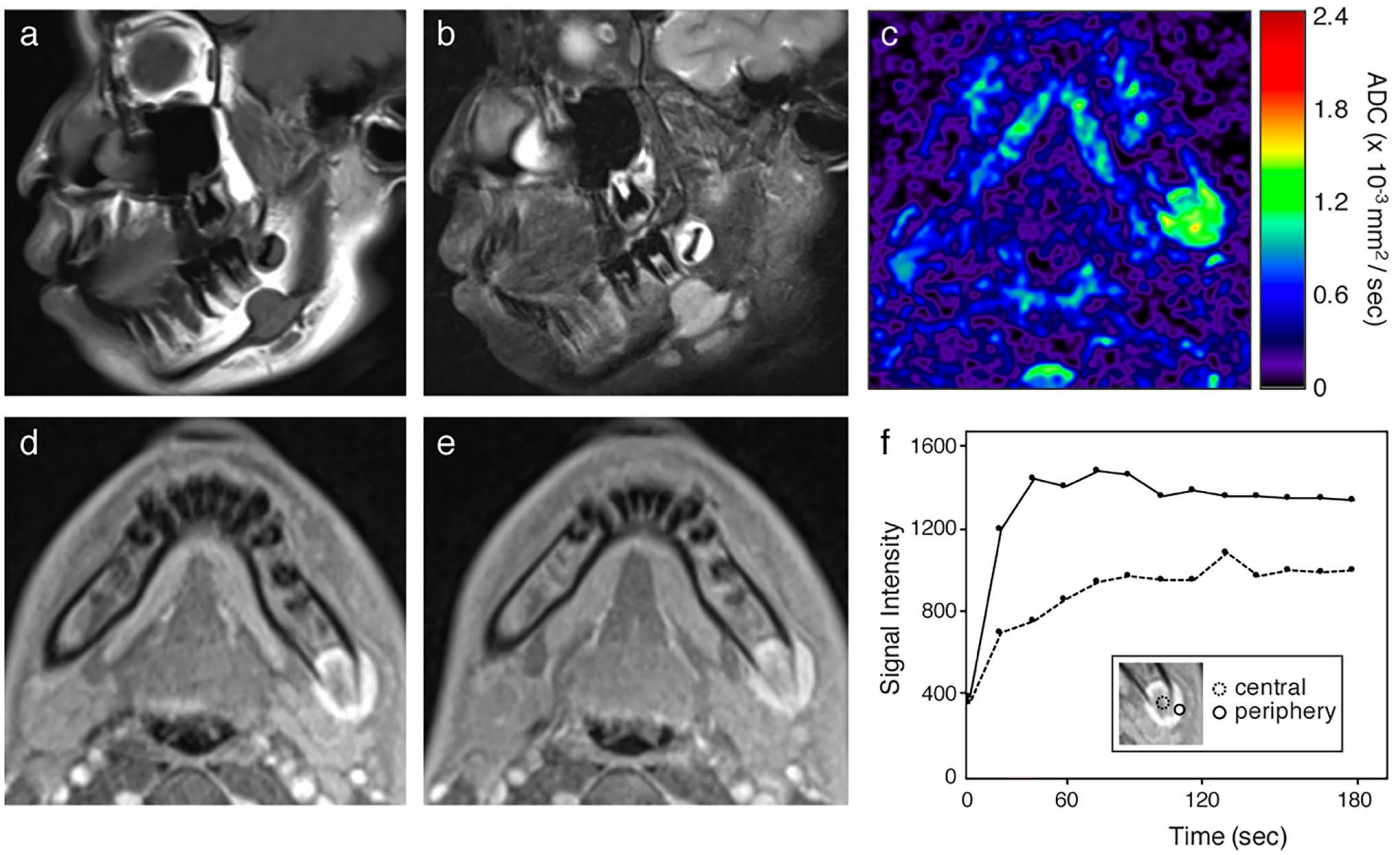
Magnetic resonance imaging of the lesion in the left mandible. **a** Sagittal T1-weighted image showing a well-defined mass with homogeneous low signal intensity. **b** Sagittal fat-suppressed T2-weighted image showing a mass with heterogeneous high signal intensity. **c** Lesion exhibiting moderate apparent diffusion coefficient (ADC; 1.2 × 10^−3^ mm^2^/s), as calculated by diffusion-weighted imaging using two b-values of 500 s/mm^2^ and 1000 s/mm^2^. **d** Dynamic contrast-enhanced image 30 s after the injection of the contrast agent. **e** Dynamic contrast-enhanced image 180 s after the injection of the contrast agent. **f** Time-intensity curves showing a gradual increment pattern in the central region of the mass and a rapid uptake followed by a gradual decrement pattern in the peripheral region of the mass

The lesion appeared as a homogeneous low signal intensity mass on T1-weighted MRI (Fig. 3a) and as a heterogeneous high signal intensity mass on fat-suppressed T2-weighted MRI (Fig. 3b). The apparent diffusion coefficient (ADC) of the lesion was calculated by diffusion-weighted imaging using two b-values of 500 s/mm^2^ and 1000 s/mm^2^ and found to be moderate (1.2 × 10^−3^ mm^2^/s) (Fig. 3c). A dynamic contrast-enhanced MRI using gadolinium revealed an increase in heterogeneous enhancement (Fig. 3d, e). The time-intensity curves showed a gradual increment pattern in the central region of the mass, whereas a pattern of rapid uptake followed by a gradual decrease was observed in the peripheral region of the mass (Fig. 3f).

**Fig. 4:**
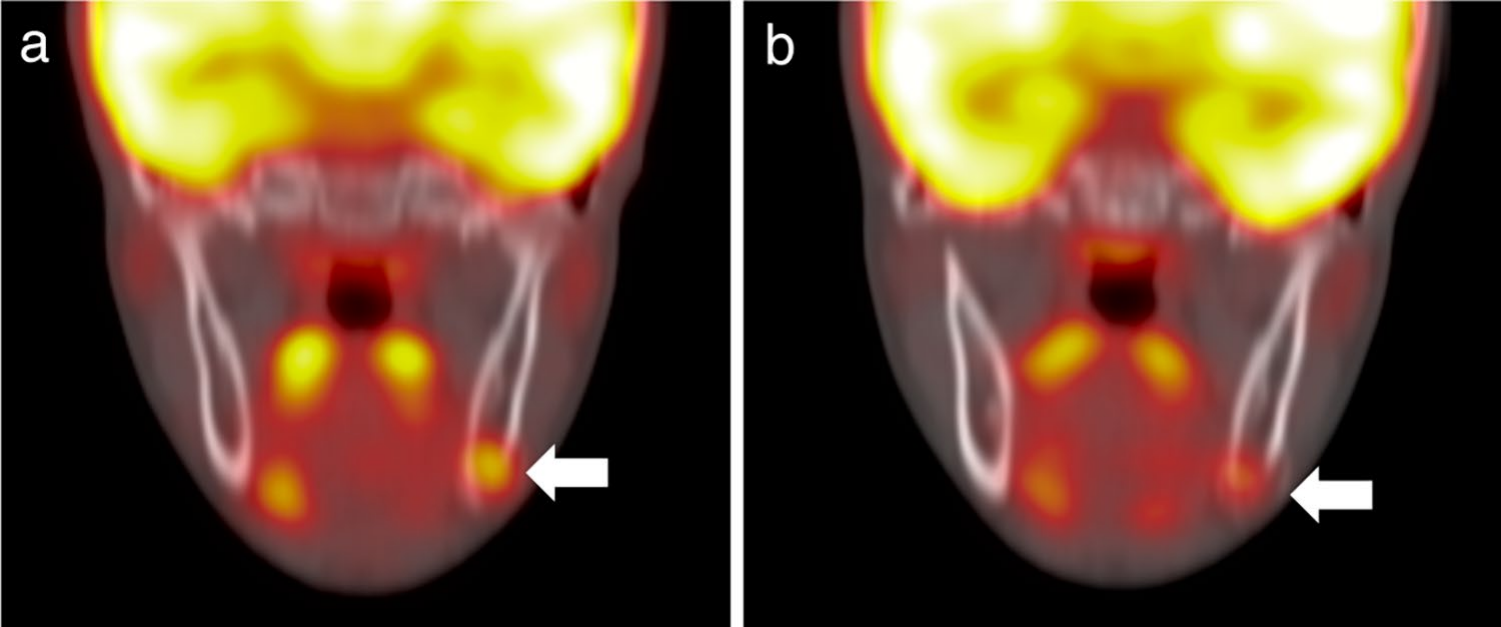
18F-fluorodeoxyglucose (FDG) positron-emission tomography/computed tomography (PET/CT) images. **a** PET/CT fusion image at 1 h after the injection of 18F-FDG showing relatively high 18F-FDG uptake in the left mandibular mass (arrow). The maximum standardized uptake value of the mass is 3.7. **b** PET/CT fusion image at 40 min after the first scan showing a decrease in 18F-FDG accumulation in the left mandibular mass (arrow). The maximum standardized uptake value of the mass is 2.6

On FDG-PET/CT, the left mandibular mass showed relatively high 18F-FDG uptake at 1 h after injection with a maximum standardized uptake value (SUVmax) of 3.7 (Fig. 4a). Interestingly, imaging at 40 min after the first scan showed a decrease in 18F-FDG accumulation in the mass to SUVmax 2.6 (Fig. 4b).

The rapidly growing hard mass with radiological findings of bone destruction and high 18F-FDG uptake was suggestive of a malignant etiology rather than a non-neoplastic or benign lesion. However, the possibility of a benign lesion could not be discarded because the radiological findings of ADC and dynamic MRI and dual-time-point FDG-PET/CT favored benign etiology, such as non-ossifying fibroma, giant cell tumor, or Langerhans cell histiocytosis.

**Fig. 5:**
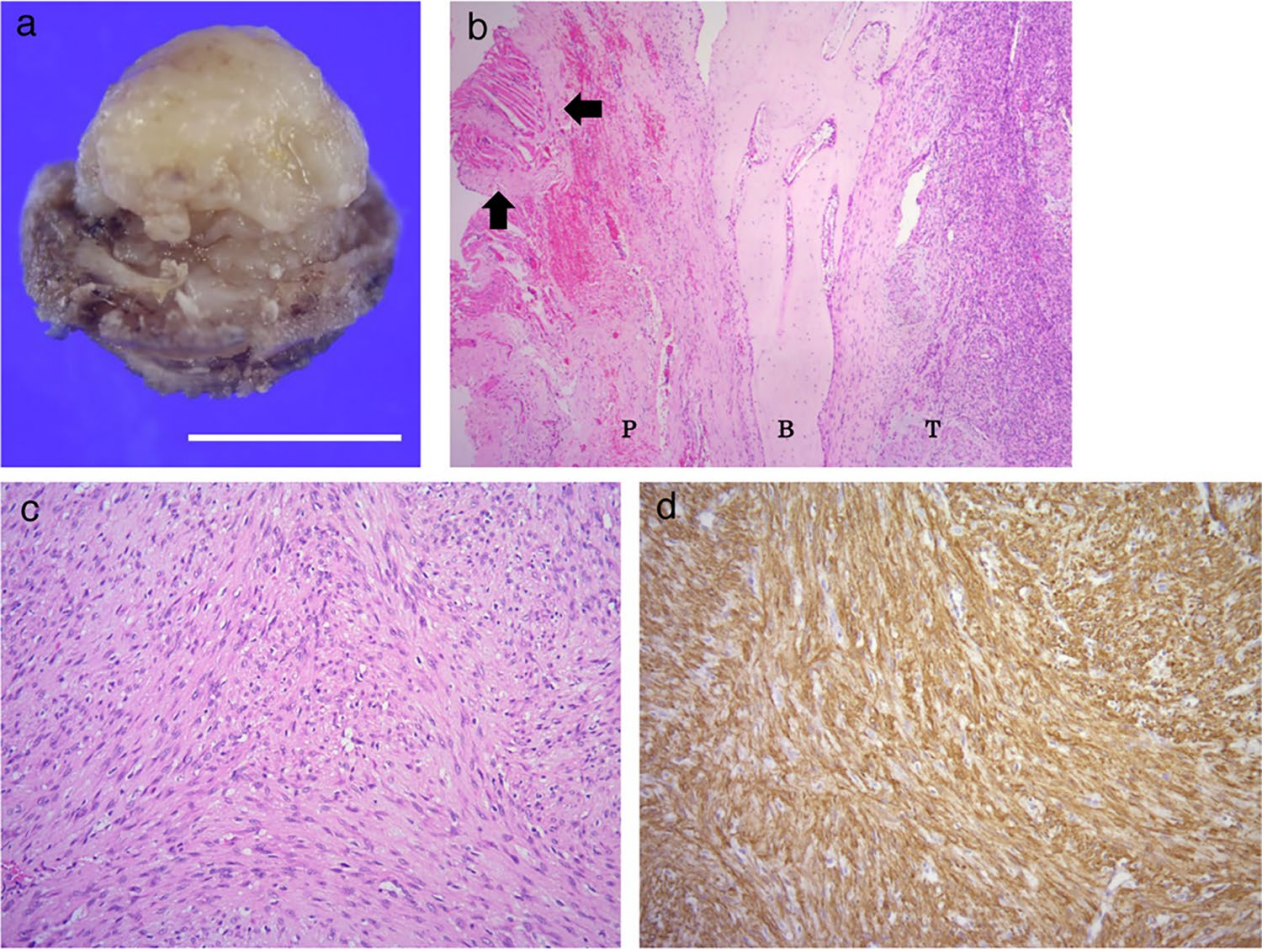
**a** Removed spherical mass. The upper half of figure is located within mandibular bone, and the lower half of figure is extrusive toward periosteum. The latter is covered with a hard connective tissue (scale bar = 1 cm). **b** Periphery of the mass. Proliferative spindle cells constitute the tumor (T). A shell-like thin bone (B) covers the tumor. Thick connective tissues including striated muscle (arrows) attached to the shell bone are observed. The connective tissue is regarded as hyperplastic periosteum (P) [stained with hematoxylin and eosin (HE)] (magnification × 4). **c** Curved and interlacing fascicles of spindle cells are observed. Cell atypia is not observed in the tumor (stained with HE) (magnification × 20). **d** Majority of tumor cells appear positive for α-SMA (immunohistochemistry for α-SMA) (magnification × 20)

A biopsy was performed one week before surgery and it was diagnosed as fibroma. The lesion was surgically excised. The extirpated spherical mass (diameter: 1.5 cm) was encompassed with the connective tissue. Bone-like hardness was partly palpable on the surface of the extrusive hemisphere toward the periosteum (Fig. 5a).

Histologically, the excised mass (i.e., lesion) was partially circumscribed with a thin shell-like bone at the periphery. Thick fibrous tissue attached to the bone surface, regarded as the periosteum, was observed (Fig. 5b). The inner portion of the mass was mainly composed of fascicles of spindle cells with bland nuclei. The tumor cell fascicles showed a curved, interlacing, and storiform pattern (Fig. 5c). The tumor contained focal myxoid matrices, slit-like vascular lumens, sparse osteoclast-like giant cells, and extravasated erythrocytes. Immunohistochemically, the tumor cells were positive for α-SMA (Fig. 5d), HHF35 and vimentin, and were negative for S100 protein, desmin, CD34 and CD68. MIB-1 (Ki-67) labeling index was 7.2%. Histological findings and immunohistochemical characteristics of myofibroblasts in tumor cells were consistent with the diagnosis of NF. Image examination revealed reactive ossification in the periosteum, which was identified as a shell-like bone in histological examination. Thus, the lesion was diagnosed as PF.

**Fig. 6:**
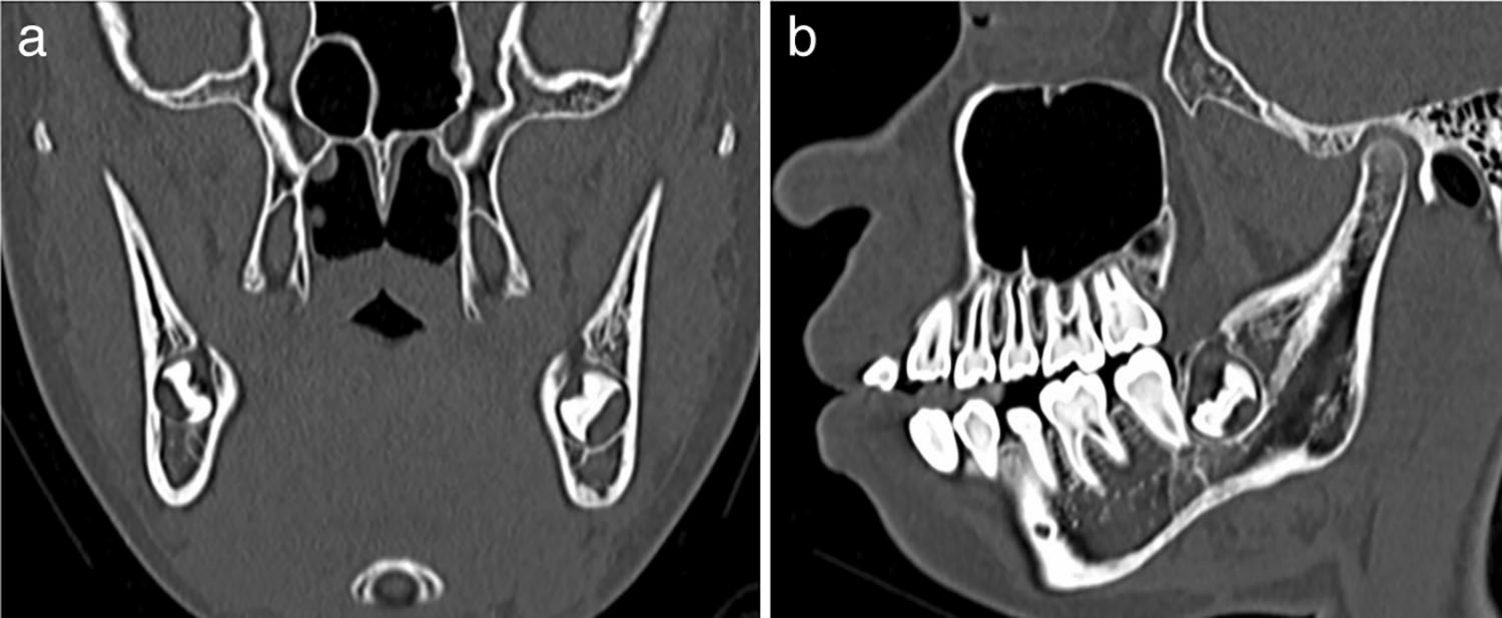
Follow-up computed tomography (CT) of the mandible at 1.5 years postoperatively showing no evidence of residual tumor or lesion recurrence with bone window settings (window width: 3000 HU; window level: 600 HU). The bone at the operated site shows complete healing. **a** Coronal CT image. **b** Sagittal CT image

The swelling of the left cheek improved after surgical excision. At 1.5 years after the operation, a CT scan was performed to re-evaluate the status of the mass. CT scan revealed that the mass had disappeared and that the bone had occupied the area (Fig. 6a, b). The patient was pain-free during recall examinations. Neither radiographic nor clinical examinations revealed any signs of mass recurrence.

## Discussion

In this report, we presented a case of PF in the mandible in a child. PF is considered a subtype of NF and is far less commonly reported. Few studies have been published on NF and/or PF in children, and notably, the occurrence of mandibular NF and/or PF in children is extremely rare. Bemrich-Stolz et al. reported 18 cases of NF in pediatric patients over a 12-year period [9]. Among them, seven patients had NF in the head and neck but none in the mandible. Recently, it has been reported that in 15 children with NF in the head and neck, the most common location of NF was the maxillofacial region, followed by the scalp, forehead, and neck. Only one patient had NF in the mandible [4].

To the best of our knowledge, in pediatric population, only three mandibular PF patients, including our case, have appeared in the literature [1, 10]. All three patients had a similar clinical feature of rapidly growing painless swelling, leading to concerns for high-grade malignant neoplasms such as sarcoma. Hence, surgical excision was performed in all three cases. Therefore, verification of the diagnosis of PF before surgery is important; however, differentiating PF from neoplastic lesions by clinical examination alone is difficult.

The radiological (CT and MRI) findings of NF and/or PF are non-specific and variable. MRI of NF shows various signal intensities, probably because of the combination of variability in cellularity, amount of collagen, amount of cytoplasm and water content in the extracellular space, and vascularity in the individual lesion [6, 11]. The relationship between signal intensities on T2-weighted MRI and histological subtypes has been advocated [2, 11, 12]. In general, the signal intensity of the lesion with myxoid or cellular histology is higher than that of muscle on T2-weighted images, whereas lesions with fibrous histology present as a markedly hypointense signal compared with the surrounding muscles on all pulse sequences. The coexistence of abundant collagen and acellularity in the fibrous lesions leads to a reduction in signal intensity on T2-weighted images [11]. In this study, the signal intensity of the mass was isointense and hyperintense compared to that of muscles on T1-weighted and T2-weighted images, respectively, favoring the predominant myxoid or cellular nature of the lesion.

Dynamic contrast-enhanced MRI using gadolinium revealed increased heterogeneous enhancement. The time-intensity curves showed a gradual increment pattern in the central region of the mass and a pattern of rapid uptake followed by a gradual decrease in the peripheral region of the mass. The time-intensity curve pattern observed in the peripheral region of the mass is similar to that of malignant tumors; however, the gradual increment pattern in the central region of the mass was atypical for malignant tumors and favored benign lesions. Also, in the present case, the ADC of the lesion was suggestive of a benign etiology rather than a malignant etiology. ADC calculated from diffusion-weighted imaging using two *b*-values of 500 s/mm^2^ and 1000 s/mm^2^ was higher than the standard value for head and neck malignancies, such as squamous cell carcinoma and malignant lymphoma [13]. However, the significance of time-intensity curves and ADC in differentiating malignant tumors from benign lesions has not been fully elucidated and requires further examinations.

FDG-PET visualizes glucose metabolism and is widely used to differentiate between benign and malignant lesions, as well as to stage/restage various malignancies. In a number of cases, the degree of 18F-FDG accumulation is used to differentiate between malignant and benign lesions. However, numerous other benign conditions, such as abscess, pulmonary granuloma, tuberculosis, and sarcoidosis, may also present with increased 18F-FDG uptake [7, 8]; thus, the role of FDG-PET in distinguishing benign or non-neoplastic lesions from malignant lesions remains controversial. Some authors have described the efficacy of dual-time-point imaging, that is serial scanning at two different (early and delayed) uptake periods, for differentiating benign lesions from malignant lesions [14, 15]. In general, 18F-FDG accumulation in malignant lesions tends to increase over several hours. On the contrary, 18F-FDG uptake by benign lesions undergoes an early plateau, providing a potential means of differentiation [16]. In the present case, dual-time-point FDG-PET/CT showed decreased 18F-FDG accumulation on the delayed scan, which favored the benign etiology over the malignant one. To the best of our knowledge, no study has evaluated PF using dual-time-point FDG-PET/CT.

The diagnosis of PF is challenging; however, the radiological characteristics of the present case, such as the time-intensity curves and ADC of MRI and dual-time-point FDG-PET/CT findings, could have potential usefulness in differentiating PF from malignancy and, thus, in the diagnosis of PF.

Because the diagnosis of PF is difficult with clinical examination and imaging, histological examination is necessary to confirm the diagnosis. Histologically, PF shows features similar to NF. Allen identified four features that are common in nearly all cases of fasciitis: (1) presence of spindle-shaped fibroblasts that tend to be arranged in long fascicles, which are slightly curved, whorled, or S-shaped; (2) presence of small vascular clefts or slit-like spaces that often separate the fibroblasts; (3) extravasation of erythrocytes; and (4) presence of mucoid interstitial ground substance [2, 12, 17]. In addition to these four features, our case revealed that the presence of a hyperplastic periosteum with reactive ossification is an important feature of PF and can be used for the diagnosis of PF. Periosteal reactions were also observed in two other PFs that occurred in the pediatric mandible [1, 10]. In general, peripheral reactions of NF other than PF are not detected histologically. NF arising from subcutis and within muscle often extend between fat cells and muscle cells, respectively [17]. Contrast to the sarcomatous infiltrating appearance in them, PF is well-circumscribed from the overlying soft tissue, and possibly encapsulated. This phenomenon is due to reactive periosteal overgrowth with reactive bone formation [12]. The mechanism of periosteal hyperplasia and bone formation is unknown but may be related with the mediators secreted from the tumor cells and/or stimulus by external force.

Once the diagnosis of NF and/or PF has been made histologically, local excision is usually the appropriate treatment. After surgical excision, the prognosis of NF and/or PF is good and the recurrence is exceedingly rare [4, 11]. In fact, two pediatric patients with mandibular PF were reported to have no signs of mass recurrence at re-evaluation. However, no radiographic images of these two cases were provided. The present report is the first to describe the CT findings at 1.5 years after surgical excision of mandibular PF in a child. Neither radiographic nor clinical examinations demonstrated any signs of mass recurrence.

In conclusion, this article describes a rare case of PF originating from the periosteum of the mandible in an 11-year-old girl who exhibited clinical and radiographic features of a neoplastic disorder. The mass was surgically excised, and the diagnosis of PF was verified histologically. PF shares common clinical and radiographic features with neoplastic mandible tumors and is often misdiagnosed as a malignant tumor of the soft tissues. Incorrect diagnosis may lead to overtreatment, potentially causing disturbed orofacial development in children. In this article, we imply the potential usefulness of MRI findings (i.e., time-intensity curves and ADC) and dual-time-point FDG-PET/CT findings to differentiate PF from malignancy. Nevertheless, in contrast to neoplastic tumors, PF has a good prognosis after surgical excision. Therefore, PF should be included in the differential diagnosis when neoplastic lesions of the mandible are encountered.
